# Exploring the association between selective serotonin reuptake inhibitors and rhabdomyolysis risk based on the FDA pharmacovigilance database

**DOI:** 10.1038/s41598-023-39482-y

**Published:** 2023-07-28

**Authors:** Yan Wang, Yajing Lin, Qing Lin, Haiming Liang, Weiming Cai, Dongbo Jiang

**Affiliations:** Department of Pharmacy, Affiliated Hospital of Guangdong Medical University, Zhanjiang, 524001 China

**Keywords:** Drug safety, Clinical pharmacology

## Abstract

Rhabdomyolysis is a syndrome potentially fatal and has been associated with selective serotonin reuptake inhibitors (SSRIs) treatment in a few case reports. Herein, we purpose to establish the correlation between SSRIs use and rhabdomyolysis using the U.S. Food and Drug Administration Adverse Event Reporting System (FAERS) database. We conducted an analysis on reports that were submitted to the FAERS database during the period between January 1, 2004, and December 31, 2022. Four algorithms, including reporting odds ratio (ROR), proportional reporting ratio (PRR), Bayesian confidence propagation neural network (BCPNN), and empirical Bayes geometric mean (EBGM), were employed to quantify the signals of rhabdomyolysis associated with SSRIs. In total, 16,011,277 non-duplicated reports were obtained and analyzed. Among 33,574 reports related to rhabdomyolysis, SSRIs were classified as primary suspected drug in 889 cases. Disproportionality analysis identified a positive signal between rhabdomyolysis and SSRIs (ROR: 2.86, 95% CI 2.67–3.05; PRR: 2.84, χ^2^: 1037.16; IC_0.25_ = 1.39; EBGM_0.5_ = 2.64). Among six SSRIs, fluvoxamine had the strongest signal (ROR: 11.64, 95% CI 8.00–16.93; PRR: 11.38, χ^2^: 265.51; IC_0.25_ = 2.41; EBGM_0.5_ = 8.31), whereas no significant signal of rhabdomyolysis was detected for paroxetine (ROR: 1.83, 95% CI 1.55–2.15; PRR: 1.82, χ^2^: 53.82; IC_0.25_ = 0.73; EBGM_0.5_ = 1.59). After excluding cases co-administered with statins, the signal of rhabdomyolysis associated with SSRIs remains significant. Our analysis reveals that there are differences in safety signals among six SSRIs in respect to the risk of rhabdomyolysis, with fluvoxamine displaying the highest risk signal, while paroxetine did not show a significant signal. Given the potentially lethal nature of rhabdomyolysis, healthcare professionals should inform patients of the potential risk of rhabdomyolysis associated with SSRIs prior to initiating treatment.

## Introduction

Rhabdomyolysis is a medical condition characterized by the destruction of skeletal muscle, leading to the release of intracellular muscle constituents such as electrolytes, enzymes, and myoglobin into the bloodstream and extracellular space^1^. The severity of rhabdomyolysis can range from an asymptomatic condition with elevated creatine phosphokinase levels to a life-threatening illness with electrolyte abnormalities, acute kidney injury (AKI) and disseminated intravascular coagulation^2^. Prompt recognition of rhabdomyolysis is critical for effective management, given that the condition has a mortality rate of approximately 10%, which is substantially higher in patients with AKI^3^. Several factors have been identified as predictors of rhabdomyolysis outcomes, including age, gender, initial creatinine levels, and the underlying cause of the condition^4^. The condition can be caused by genetic defects underlying metabolic myopathies or by a wide variety of acquired factors such as drugs and toxins, infections, physical trauma, and exertional stress^5^. Drug-induced rhabdomyolysis is a significant form of rhabdomyolysis and can be caused by over 200 different drugs, making it an idiosyncratic reaction^6^. This underscores the importance of careful monitoring for signs of rhabdomyolysis in patients receiving any medication that may be associated with the condition.

Since fluoxetine was first introduced to the U.S. in 1988, selective serotonin reuptake inhibitors (SSRIs) have been a fundamental part of psychopharmacology for more than three decades^7^. Currently, there are mainly six SSRIs marketed worldwide, including fluoxetine, citalopram, escitalopram, paroxetine, sertraline and fluvoxamine, which have definite curative effects for treating major depressive disorder. Despite being generally better tolerated than other antidepressants, SSRIs have been associated with several less common adverse drug reactions (ADRs) such as serotonin syndrome, QT prolongation, and suicidal ideation^8^. Meanwhile, several case reports have linked the occurrence of rhabdomyolysis with the use of SSRIs^9–14^. As SSRIs are widely used, it is crucial to comprehend the association between these medications and rhabdomyolysis in order to effectively minimize the risk of death due to this condition in patients who are taking them. Nonetheless, the occurrence of SSRIs-induced rhabdomyolysis is low, which poses a challenge in gathering trustworthy evidence from clinical trials with limited sample sizes. To address this, conducting a post-marketing pharmacovigilance study may be a practical approach to reveal such evidence.

The FDA Adverse Events Reporting System (FAERS) is a database comprising of spontaneous adverse event reports that are submitted to FDA, which is designed to assist the FDA in its post-marketing safety surveillance efforts for therapeutic biologic products and drugs^15^. The database consists of more than 26 million reports since 1969 to 2022. Disproportionality analysis is a widely used pharmacovigilance method to identify safety signals for drugs within spontaneous adverse event reporting databases^16^. Therefore, we performed a disproportionality analysis based on the FAERS database to characterize and evaluate rhabdomyolysis associated with SSRIs.

## Results

### Basic information of cases

**Figure 1:**
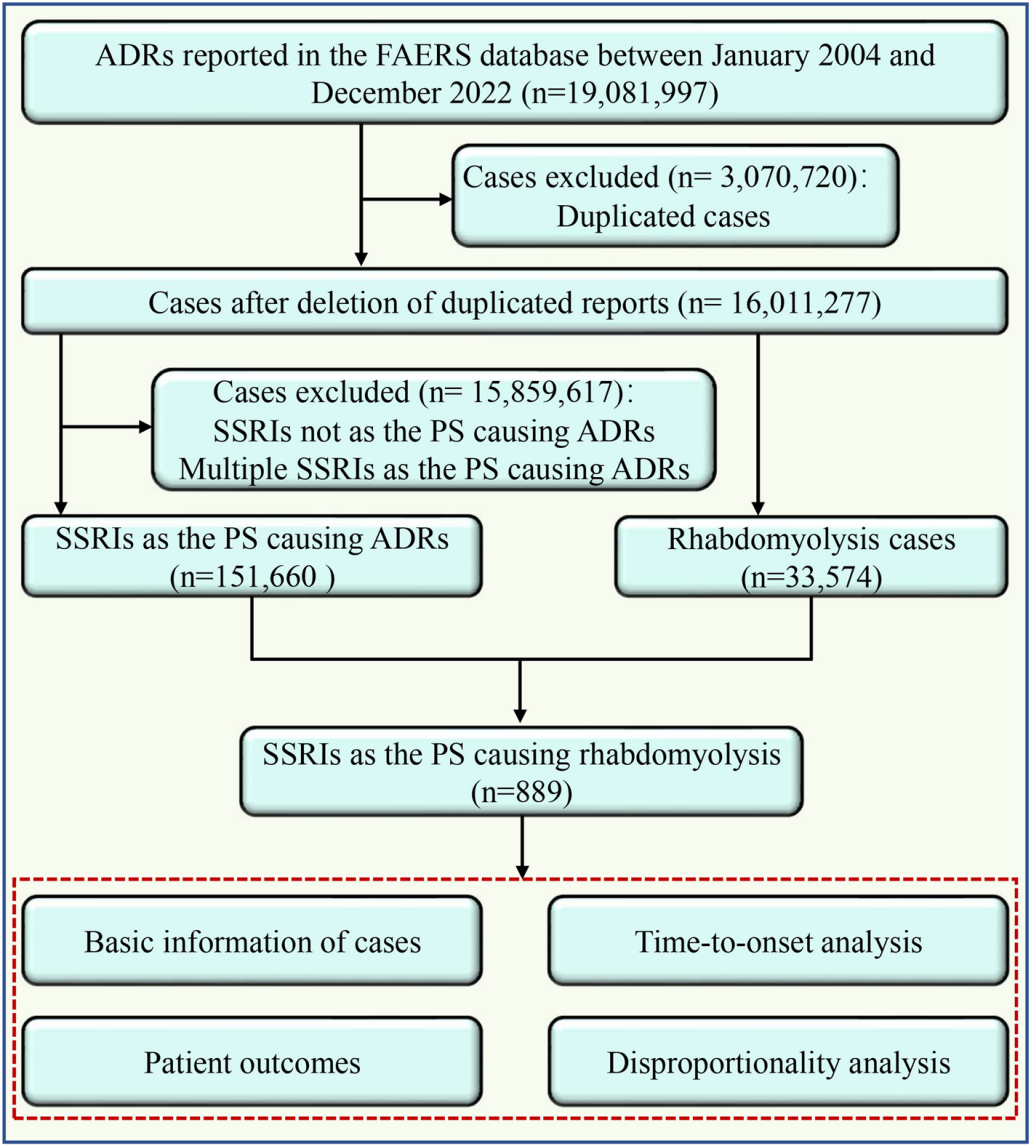
The flow chart of this work.

The flow diagram of this study is illustrated in Fig. 1. A total of 16,011,277 non-duplicated cases submitted between January 2004 and December 2022 were obtained from the FAERS database. Among them, there were 33,574 cases of rhabdomyolysis and 151,660 reports recognized SSRIs as the PS causing ADRs. Finally, a total of 889 cases were identified which SSRIs were recognized as the PS causing rhabdomyolysis, including 288 cases of sertraline, 2163 cases of fluoxetine, and 30 cases of 145 cases of paroxetine, 136 cases of escitalopram, 129 cases of citalopram and 28 cases of fluvoxamine. As shown in Table 1, sertraline had the highest proportion of SSRIs-related rhabdomyolysis reports (32.40%), followed by fluoxetine (18.34%) and paroxetine (16.31%). Of all the included cases, the majority (56.92%) were between the ages of 18 and 65, with a median age of 39.00 years. Among all the cases with known gender, the proportion of female cases (46.57%) was slightly higher than that of male cases (43.76%). However, in cases related to fluvoxamine, the proportion of male cases was markedly higher than that of female cases. Health professionals, including physicians, pharmacists and other health professionals, submitted 82% of the reports. Among the reporting countries, the highest number of cases (24.18%) was reported from the USA, followed by France (11.70%), the UK (11.25%), Italy (8.77%), and Japan (8.66%).

**Table 1:** Demographic and administrative characteristics of patients with SSRIs-associated rhabdomyolysis.

Characteristics	SSRIs *N* = 889	Sertraline *N* = 288	Fluoxetine *N* = 163	Paroxetine *N* = 145	Escitalopram *N* = 136	Citalopram *N* = 129	Fluvoxamine *N* = 28
Age [Median (Q1–Q3)] (years)	39.00 [25.00, 62.00]	36.00 [22.00, 55.00]	25.00 [20.25, 52.75]	59.50 [39.25, 71.00]	32.50 [25.00, 63.00]	47.00 [33.50, 58.00]	36.00 [17.00, 51.00]
Age group [n (%)]							
< 18 years	93 (10.46)	46 (15.97)	30 (18.40)	2 (1.38)	3 (2.21)	4 (3.10)	8 (28.57)
18–65 years	506 (56.92)	150 (52.08)	102 (62.58)	72 (49.66)	81 (59.56)	90 (69.77)	11 (39.29)
> 65 years	153 (17.21)	46 (15.97)	14 (8.59)	52 (35.86)	24 (17.65)	13 (10.08)	4 (14.29)
Unknown	137 (15.41)	46 (15.97)	17 (10.43)	19 (13.10)	28 (20.59)	22 (17.05)	5 (17.86)
Gender [n (%)]							
Female	414 (46.57)	160 (55.56)	56 (34.36)	72 (49.66)	58 (42.65)	63 (48.84)	5 (17.86)
Male	389 (43.76)	99 (34.38)	96 (58.90)	58 (40.00)	65 (47.79)	54 (41.86)	17 (60.71)
Unknown	86 (9.67)	29 (10.07)	11 (6.75)	15 (10.34)	13 (9.56)	12 (9.30)	6 (21.43)
Reporter [n (%)]							
Health-professional	729 (82.00)	259 (89.93)	129 (79.14)	91 (62.76)	118 (86.76)	114 (88.37)	18 (64.29)
Consumer/lawyer	102 (11.47)	10 (3.47)	22 (13.50)	48 (33.10)	13 (9.56)	8 (6.20)	1 (3.57)
Unknown	58 (6.52)	19 (6.60)	12 (7.36)	6 (4.14)	5 (3.68)	7 (5.43)	9 (32.14)
Reporting country [n (%)]	40 (4.50)	15 (5.21)	2 (1.23)	11 (7.59)	5 (3.68)	5 (3.88)	2 (7.14)
USA	215 (24.18)	64 (22.22)	60 (36.81)	25 (17.24)	34 (25.00)	24 (18.60)	8 (28.57)
France	104 (11.70)	22 (7.64)	24 (14.72)	26 (17.93)	18 (13.24)	14 (10.85)	0 (0.00)
UK	100 (11.25)	25 (8.68)	32 (19.63)	21 (14.48)	0 (0.00)	22 (17.05)	0 (0.00)
Italy	78 (8.77)	52 (18.06)	7 (4.29)	0 (0.00)	11 (8.09)	8 (6.20)	0 (0.00)
Japan	77 (8.66)	15 (5.21)	3 (1.84)	38 (26.21)	9 (6.62)	1 (0.78)	11 (39.29)

### Time-to-onset analysis

**Figure 2:**
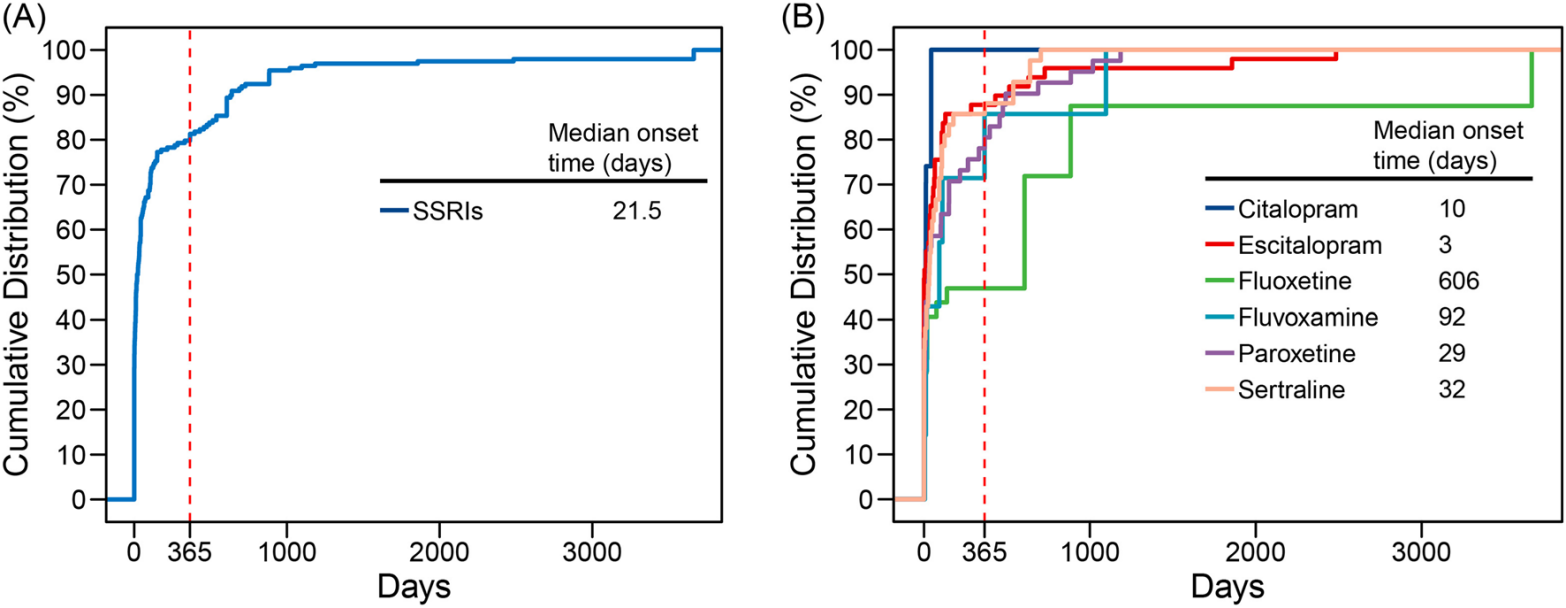
The time-to-onset of rhabdomyolysis related to (**A**) all SSRIs and (**B**) single SSRIs.

The time-to-onset of rhabdomyolysis was calculated as the duration between the START_DT in the THER files and the EVENT_DT in the DEMO files. Cases with input errors, including those where the EVENT_DT preceded the START_DT and those with inaccurate dates, were excluded from the analysis. Ultimately, a total of 198 cases met the inclusion criteria and were included in the time-to-onset analysis. SSRIs-associated rhabdomyolysis had a median onset time of 21.5 days (Q1–Q3: 0–135.75), with 79.80% of cases occurring within a 1-year period (Fig. 2A). The median time-to-onset for escitalopram was 3 days (Q1–Q3: 0–65), for citalopram was 10 days (Q1–Q3: 0–27.5), for paroxetine was 29 days (Q1–Q3: 2–265), for sertraline was 32 days (Q1–Q3: 0–107), for fluvoxamine was 92 days (Q1–Q3: 14–238), and for fluoxetine was 606 days (Q1–Q3: 4.75–884) (Fig. 2B).

### Outcomes of patients with SSRIs-associated rhabdomyolysis

**Figure 3:**
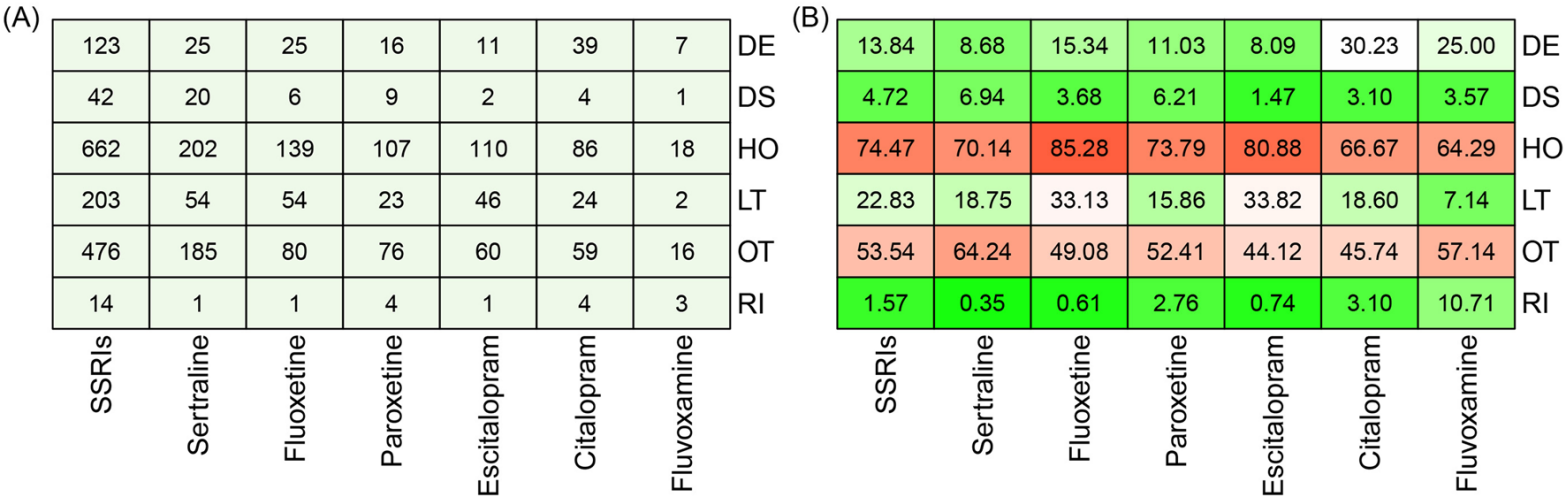
The number (**A**) and proportion (**B**) of serious outcomes observed in cases of SSRIs-associated rhabdomyolysis. DE, Death; DS, Disability; HO, Hospitalization; LT, life-threatening; OT, Other Serious; RT, Required intervention to prevent permanent impairment.

We conducted a detailed assessment of cases with SSRIs-associated rhabdomyolysis by examining the occurrence of serious outcomes, including death, hospitalization, life-threatening situations, disabilities, required intervention to prevent permanent impairment, and other serious outcomes. As shown in Fig. 3, the mortality rate, life-threatening rate, and hospitalization rate of cases with SSRIs-associated rhabdomyolysis were 13.84%, 22.83%, and 74.47%, respectively. Among the six SSRIs, the citalopram group had the highest mortality rate of 30.23%, followed by the fluvoxamine and fluoxetine groups. The escitalopram group had the highest rate of life-threatening conditions, with a rate of 33.82%, followed by the fluoxetine and sertraline groups. The sertraline group had the highest hospitalization rate, reaching 85.28%, while the hospitalization rates in the other groups were all over 60%.

### Disproportionality analysis

According to disproportionality analysis, the use of SSRIs is associated with an increased risk of rhabdomyolysis as all four algorithms fulfilled their respective criteria (ROR: 2.86, 95% CI 2.67–3.05; PRR: 2.84, χ^2^: 1037.16; IC_0.25_ = 1.39; EBGM_0.5_ = 2.64) (Table 2). For individual SSRIs, fluvoxamine exhibited a strongest risk signal (ROR: 11.64, 95% CI 8.00–16.93; PRR: 11.38, χ^2^: 265.51; IC_0.25_ = 2.41; EBGM_0.5_ = 8.31), followed by fluoxetine (ROR: 3.49, 95% CI 2.99–4.07; PRR: 3.47, χ^2^: 285.85; IC_0.25_ = 1.53; EBGM_0.5_ = 3.04) and escitalopram (ROR: 3.39, 95% CI 2.86–4.01; PRR: 3.37, χ^2^: 226.51; IC_0.25_ = 1.48; EBGM_0.5_ = 2.92). However, the absence of significant signal in paroxetine was indicated as the PRR and EBGM05 values did not meet their corresponding statistical criteria (ROR: 1.83, 95% CI 1.55–2.15; PRR: 1.82, χ^2^: 53.82; IC_0.25_ = 0.73; EBGM_0.5_ = 1.59). Due to the well-established evidence of the association between statins and rhabdomyolysis, we are considering whether the positive signal between SSRIs and rhabdomyolysis could be attributed to the co-administration of statins. Therefore, we excluded cases co-administered with statins and conducted further analysis. Among the 151,660 cases recognized with SSRIs as the PS causing ADRs, 7780 cases involved co-administration of statins. After excluding these cases, there were a total of 800 SSRIs-associated rhabdomyolysis cases that did not involve concomitant use of statins. The disproportionality analysis demonstrates that even after excluding such cases, the signal of rhabdomyolysis associated with SSRIs remains significant (ROR: 2.70, 95% CI 2.52–2.90; PRR: 2.69, χ^2^: 832.21; IC_0.25_ = 1.31; EBGM_0.5_ = 2.50) (Table 3).

**Table 2:** Signal detection for SSRIs-associated rhabdomyolysis.

Drugs	ROR (95% CI)	PRR (χ^2^)	IC (IC_0.25_)	EBGM (EBGM_0.5_)
SSRIs (*N* = 889)	2.86 (2.67, 3.05)	2.84 (1037.16)	1.48 (1.39)	2.80 (2.64)
Sertraline (*N* = 288)	2.94 (2.62, 3.30)	2.93 (363.08)	1.54 (1.37)	2.91 (2.64)
Fluoxetine (*N* = 163)	3.49 (2.99, 4.07)	3.47 (285.85)	1.79 (1.53)	3.46 (3.04)
Paroxetine (*N* = 145)	1.83 (1.55, 2.15)	1.82 (53.82)	0.86 (0.73)	1.82 (1.59)
Escitalopram (*N* = 136)	3.39 (2.86, 4.01)	3.37 (226.51)	1.75 (1.48)	3.36 (2.92)
Citalopram (*N* = 129)	2.63 (2.21, 3.13)	2.62 (128.91)	1.39 (1.17)	2.61 (2.26)
Fluvoxamine (*N* = 28)	11.64 (8.00, 16.93)	11.38 (265.51)	3.51 (2.41)	11.37 (8.31)

**Table 3:** Signal detection for SSRIs-associated rhabdomyolysis after excluding cases co-administered with statins.

Drugs	ROR (95% CI)	PRR (χ^2^)	IC (IC_0.25_)	EBGM (EBGM_0.5_)
SSRIs (*N* = 800)	2.70 (2.52, 2.90)	2.69 (832.21)	1.41 (1.31)	2.65 (2.50)
Sertraline (*N* = 248)	2.67 (2.35, 3.03)	2.66 (255.47)	1.4 (1.24)	2.65 (2.38)
Fluoxetine (*N* = 156)	3.50 (2.98, 4.09)	3.48 (274.61)	1.79 (1.53)	3.47 (3.04)
Paroxetine (*N* = 132)	1.73 (1.46, 2.05)	1.73 (40.32)	0.79 (0.66)	1.72 (1.49)
Escitalopram (*N* = 124)	3.30 (2.76, 3.93)	3.28 (196.24)	1.71 (1.43)	3.27 (2.82)
Citalopram (*N* = 112)	2.44 (2.02, 2.94)	2.43 (94.18)	1.28 (1.06)	2.43 (2.08)
Fluvoxamine (*N* = 28)	12.06 (8.28, 17.55)	11.78 (276.68)	3.56 (2.44)	11.78 (8.6)

## Discussion

Post-marketing surveillance is necessary to monitor the safety and effectiveness of pharmaceutical and biologic products after they have been approved by regulatory agencies such as the FDA. This is important because clinical trials, which are conducted prior to approval, involve a relatively small and select group of patients and may not detect all possible adverse effects associated with the product's use in a larger population. Post-marketing surveillance enables the identification and evaluation of adverse effects that were not detected during the pre-approval stage and allows for prompt action to be taken to protect public health. The FAERS database serves as a vital resource for post-marketing surveillance, allowing researchers to access it freely for pharmacovigilance and pharmacoepidemiologic signal detection studies.

In 2019, the worldwide consumption of SSRIs surpassed that of all other types of antidepressants combined, making them the most widely used antidepressants^17^. This is due to their favorable risk–benefit ratio, which has led to their recommendation as a first-line treatment for psychiatric conditions such as depression and generalized anxiety disorders. The growing number of patients with depression, as well as the surge in psychological disorders associated with the COVID-19 pandemic^18^, are expected to lead to a continued increase in the usage of SSRIs in the future. In light of this trend, it is important to conduct post-marketing surveillance studies to ensure the safety of these drugs. Previous studies have used the FAERS database to elucidate the relationship between SSRIs and suicidality^19^, mania^20^, and postpartum bleeding^21^. Some case reports have suggested that using SSRIs could lead to rhabdomyolysis, a potentially life-threatening syndrome. However, there has not been any comprehensive investigation involving larger patient populations to determine the risk of rhabdomyolysis following the use of SSRIs.

Our analysis of the FAERS database identified 889 cases of rhabdomyolysis associated with the use of SSRIs. The median age of individuals experiencing rhabdomyolysis due to SSRIs was 39.00 years, which aligns with previous research indicating a higher incidence of rhabdomyolysis among patients aged 18–65 years. Specifically, a retrospective cohort study involving 2371 patients found a mean age of 50.7 years among those with rhabdomyolysis^4^. In a multicenter retrospective study involving 387 patients with severe rhabdomyolysis, the median age was found to be 49 years^22^. There was no significant difference in the occurrence rates of SSRIs-associated rhabdomyolysis between females and males. In terms of reporters, 82% of the reports were submitted by healthcare professionals. This may be due to the fact that rhabdomyolysis is a complex disease that requires a systematic assessment by healthcare professionals to make a diagnosis. Furthermore, the fact that 74.47% of hospitalizations due to SSRIs-associated rhabdomyolysis indicates that this condition requires diagnosis and treatment by healthcare professionals in a hospital setting. The fact that the top five reporting countries were all developed nations—USA, France, UK, Italy, and Japan—can be explained in two ways. Firstly, since the FAERS database is an English-language database developed by the FDA in the USA, it is not surprising that the majority of reports come from the USA and other countries where English is the primary language. Secondly, pharmacovigilance is still a relatively new concept and may not be given high priority in developing countries^23^, which could explain why developing countries have lower reporting rates.

The median interval between the start of SSRIs treatment and the onset of rhabdomyolysis was 23.5 days, with a range of 0–135.75 days. Notably, 29.29% of cases experienced rhabdomyolysis on the same day after initiating SSRIs treatment, while 79.80% of cases occurred within the first year of treatment. However, there have been several cases of rhabdomyolysis manifesting up to a decade after the administration of SSRIs, making it difficult to assess the temporal relationship between the two from a pharmacokinetic perspective^11^. Previous studies have reported cases of sertraline-induced rhabdomyolysis after 3 months of therapy^24^, escitalopram-induced rhabdomyolysis after 2 months of therapy^10^, and pravastatin-induced rhabdomyolysis after more than 3 years of therapy^25^. These cases, along with our study, indicate that the risk of drug-induced rhabdomyolysis may persist even after long term use of certain medications. Therefore, it is crucial to continuously monitor patients and remain vigilant for symptoms such as muscle pain, weakness, and dark urine during treatment with SSRIs to identify potential cases of rhabdomyolysis.

The death rate for cases with SSRIs-associated rhabdomyolysis in our study was 13.84%, which is similar to the 12% death rate for rhabdomyolysis reported in all ADRs received by the FAERS during 2017^26^. Actually, the mortality rates reported for rhabdomyolysis exhibit a wide range of variation, from 3.4 to 59%, depending on factors such as the characteristics of the study population and setting, as well as the severity and number of coexisting conditions^27^. Therefore, the reason for the different death rates of rhabdomyolysis caused by different SSRIs in our study may be due to differences in the populations taking the medications. However, this requires more cases in the future to investigate population differences in rhabdomyolysis caused by different SSRIs. Patients who experience SSRIs-associated rhabdomyolysis have a high rate of hospitalization primarily because rhabdomyolysis is a medical emergency that often needs hospital admission to receive extracellular volume expansion to prevent AKI. A prior study found that individuals with rhabdomyolysis had a median hospitalization period of 13 days, and the duration of hospitalization was even longer for those who developed AKI.

The results of disproportionality analysis in our work showed that significant signals were detected between rhabdomyolysis and SSRIs treatment. Further analysis confirmed the previously known associations of rhabdomyolysis with escitalopram, fluoxetine, sertraline, citalopram and fluvoxamine as reported in case reports^10–14^. However, our analysis did not find sufficient evidence to suggest a significant signal of paroxetine-associated rhabdomyolysis. On the other hand, fluvoxamine showed the strongest risk signal with an ROR of 11.64. However, it is worth noting that the number of reported cases of fluvoxamine-associated rhabdomyolysis is limited, and further cases are required to verify this finding. Furthermore, while there have been reports of various drugs potentially causing rhabdomyolysis, these are often isolated cases without a well-established association. In contrast, clinical trials have widely demonstrated a clear link between statins and rhabdomyolysis^28^. In our work, after excluding cases where SSRIs were co-administered with statins, we still observed a significant signal of rhabdomyolysis associated with SSRIs. This finding indicates that SSRIs alone can be linked to an increased risk of rhabdomyolysis, independent of statin use.

The precise mechanism by which SSRIs can cause rhabdomyolysis is not yet fully understood. However, both human and rodent studies have revealed that SSRIs affect functional, structural and metabolic properties in skeletal muscle tissue^29^. The SSRIs function by inhibiting the reuptake of serotonin, which leads to an elevation in the concentration of serotonin in the synaptic cleft. Research has shown that rhabdomyolysis is one of the complications of serotonin toxicity^30^. It has been reported that 5-HT receptors have important roles in drug-induced rhabdomyolysis and other serotonin toxicity related symptoms. Treatment of zebrafish larvae with an agonist of the 5-HT_2A_ receptor resulted in a decrease in muscle birefringence and reduced immunostaining for myoseptal and myofibril proteins in skeletal muscle, which were consistent with rhabdomyolysis^31^. Additionally, rhabdomyolysis induced by the serotonin receptor agonist could be prevented by treatment with either a 5-HT_2A_ antagonist or a 5-HT_2C_ antagonist^32^. Furthermore, activation of the 5-HT_2A_ receptor by serotonin or other agonists results in the release of calcium from the intracellular stores, through the coupling of the receptor to a G-protein^33^. Elevated intracellular calcium levels lead to skeletal muscle cell death by activating proteases, intensifying contractility, inducing mitochondrial dysfunction, and increasing reactive oxygen species production^34^. While these studies offer some understanding of how SSRIs may cause rhabdomyolysis, more research is needed to investigate the mechanisms underlying this association.

In conclusion, a disproportionality analysis based on the FAERS has provided additional evidence to support previous case reports, indicating a significant association between certain SSRIs and rhabdomyolysis. Our analysis demonstrates that there are differences in the safety signals of SSRIs regarding the risk of rhabdomyolysis, with fluvoxamine exhibiting the strongest risk signal while paroxetine does not display a significant signal. Therefore, healthcare providers should consider these differences when choosing appropriate medications and inform patients of the potential risk of rhabdomyolysis, especially in those with pre-existing muscle disease or taking medications that may increase the risk of rhabdomyolysis. However, further research is needed to explore the potential mechanisms and risk factors underlying the SSRIs-associated rhabdomyolysis.

There are certain limitations that must be acknowledged in the present work like other pharmacovigilance studies based on the FAERS database. Firstly, given that the FAERS operates as a database for spontaneously reported adverse events, there is a risk of both under-reporting and misreporting. For example, misspelling the name of drugs could lead to this study missing some cases, while inaccuracies in the recording of START_DT and EVENT_DT could result in biases in the time-to-onset results. Secondly, it is widely recognized that the FAERS dataset contains instances of duplicate reports and substantial amounts of missing data. To mitigate this issue, in our analysis, we excluded some duplicate reports by using the CASEID. However, there are also duplicate reports which the same case was submitted by different reporters, resulting in different CASEIDs assigned. Due to the lack of specific characteristics in these reports, it becomes challenging to identify and deduplicate them. Thirdly, a case report may include multiple drugs, which means that cases of SSRIs-associated rhabdomyolysis may involve drugs other than SSRIs. Therefore, to increase the reliability of this study, we only included reports which SSRIs were identified as the PS, and utilized four algorithms to determine the association between SSRIs and rhabdomyolysis. Furthermore, we further strengthened the reliability of our conclusion by conducting an additional analysis after excluding statins, which are the most likely drugs to cause rhabdomyolysis. Lastly, the unavailability of data on the total number of patients using SSRIs makes it difficult to accurately calculate the exact incidence and mortality of SSRIs-associated rhabdomyolysis. Nevertheless, since SSRIs-related rhabdomyolysis is relatively uncommon, our analysis of large database may enhance the level of confidence regarding the association between the use of SSRIs and rhabdomyolysis. This can provide valuable evidence for further research and clinical practice in this field.

## Methods

### Data source

Data were retrieved based on the FAERS database from the first quarter (Q1) of 2004 to the fourth quarter (Q4) of 2022 (https://fis.fda.gov/extensions/FPD-QDE-FAERS/FPD-QDE-FAERS.html). The FAERS data files contain demographic and administrative information (DEMO), drug information (DRUG), indications for use (INDI), patient outcomes (OUTC), adverse drug reaction information (REAC), therapy start dates and end dates for reported drugs (THER), and report sources (RPSR).

### Screening of rhabdomyolysis cases related to SSRIs

To screen for cases of rhabdomyolysis associated with SSRIs, we conducted a four-step analysis. First, we consolidated all relevant reports and removed any duplicates based on FDA-recommended methods. Specifically, we used the most recent FDA_DT when the CASEID was the same, and we chose the highest PRIMARYID when both CASEID and FDA_DT were identical. Next, we screened ADR reports for each SSRI by matching the generic and brand names of the corresponding drugs in the DRUG file. The FAERS categorizes the role of each drug in its associated ADRs as Primary Suspect (PS), Secondary Suspect (SS), Concomitant (C), or Interacting (I). If a drug is classified as PS, it means that the drug is the most likely cause of the adverse drug reaction among all medications taken by the patient. Therefore, to focus our results specifically on the drug most likely responsible for rhabdomyolysis, we restricted our analysis to reports where SSRIs were considered as the PS. We excluded cases in which multiple types of SSRIs were categorized as the PS. Thirdly, we extracted all cases of rhabdomyolysis from the REAC files, using the preferred term "rhabdomyolysis". Finally, we identified all cases of rhabdomyolysis related to the use of SSRIs by taking the intersection of CASEID between the rhabdomyolysis cases and the cases of SSRIs as the PS causing ADRs.

### Statistical analysis

The statistical analysis for this study was conducted using R software version 4.2.0. Descriptive analysis was used to summarize the demographic and administrative characteristics of SSRIs-associated rhabdomyolysis. Disproportionality analysis are based on a two-by-two contingency table (Table 4).

**Table 4:** A 2 × 2 contingency table for disproportionality analysis.

	ADR of interest	All other ADRs	Total
Drug of interest	a	b	a + b
All other drugs	c	d	c + d
Total	a + c	b + d	a + b + c + d

To measure the association between the use of SSRIs and rhabdomyolysis, four statistical algorithms commonly used in disproportionality analysis were employed: reporting odds ratio (ROR), proportional reporting ratio (PRR), Bayesian confidence propagation neural network (BCPNN), and empirical Bayes geometric mean (EBGM). The equations and criteria for these algorithms are presented in Table 5. Previously, Park et al.^35^evaluated various data mining methods for signal detection and found that no single method outperformed the others across all performance measures. They recommended using multiple methods and making decisions based on their collective results for drug‒adverse event surveillance. Accordingly, in our work, a safety signal was considered significant only if all four algorithms met their respective criteria.

**Table 5:** Pharmacovigilance signal detection algorithms and criteria.

Algorithms	Equations	Criteria
ROR	ROR = (a/b)/(c/b) 95%CI = exp(ln(ROR) ± 1.96 $\sqrt{\text{1/a+1/b+1/c+1/d}}$)	95%CI > 1, N ≥ 2
PRR	PRR = (a/(a + c))/(b/(b + d)	PRR ≥ 2, χ^2^ ≥ 4, N ≥ 3
BCPNN	IC = log_2_a(a + b + c + d)/((a + c)(a + b)) IC025 = exp(ln(IC)-1.96 $\sqrt{\text{1/a+1/b+1/c+1/d}}$)	IC025 ≥ 0
EBGM	EBGM = a(a + b + c + d)/ ((a + c)(a + b)) EBGM05 = exp(ln(EBGM)-1.64 $\sqrt{\text{1/a+1/b+1/c+1/d}}$)	EBGM05 > 2

ROR, Reporting odds ratio; CI, Confidence; N, Number of reports; PRR, Proportional reporting ratio; χ^2^, Chi-squared; BCPNN, Bayesian confidence propagation neural network; IC, Information component; IC025, The lower limit of the 95% two-sided CI of the IC. EBGM, Empiric Bayes geometric mean; EBGM05 and the lower 90% one-sided CI of EBGM.

## Data Availability

The raw data included in this work were downloaded from the FAERS database at https://fis.fda.gov/extensions/FPD-QDE-FAERS/FPD-QDE-FAERS.html.
